# Acute Mesenteric Ischemia Prior to Emergency Cardiac Surgery for Infective Endocarditis: Can We Design a Strategy to Improve Outcomes?

**DOI:** 10.7759/cureus.24532

**Published:** 2022-04-27

**Authors:** Héctor González-Pacheco, Rodrigo Gopar-Nieto, Adriana Torres-Machorro, Pablo E Pérez-Pinetta, Alexandra Arias-Mendoza

**Affiliations:** 1 Coronary Care Unit, National Institute of Cardiology, Mexico City, MEX; 2 Department of Cardiovascular Surgery, National Institute of Cardiology, Mexico City, MEX

**Keywords:** septic embolism, endovascular treatment, emergency cardiac surgery, infectious endocarditis, acute mesenteric ischemia

## Abstract

Infective endocarditis (IE) can be complicated by systemic embolization. Unfortunately, in some situations, it requires radical and urgent therapeutic approaches. Herein, we describe a case of IE complicated by acute mesenteric ischemia (AMI) due to septic embolism prior to emergent cardiac surgery. A previously healthy 38-year-old woman was admitted to our emergency department with a diagnosis of mitral valve IE. She presented with tachycardia and was tachypneic. In addition, a systolic murmur in the mitral area and Janeway lesions were documented. Transthoracic and transesophageal echocardiography confirmed large mobile vegetations on the mitral valve and the presence of mitral regurgitation. A thoracic computed tomography scan showed splenic and bilateral renal infarctions. Emergency mitral valve replacement was scheduled. Prior to surgery, AMI developed because of occlusion of the superior mesenteric artery (SMA). Endovascular treatment was performed with percutaneous aspiration, thrombectomy, and in situ fibrinolysis, yielding satisfactory results. Ten hours later, she underwent cardiac surgery. AMI developed postoperatively due to re-occlusion of the SMA, requiring an open laparotomy with mesenteric revascularization and extensive resection of the necrotic bowel. The patient died 18 days after hospitalization. In the IE setting, AMI is a very rare, potentially life-threatening complication. This case highlights the importance of recognizing this complication and designing a better therapeutic strategy to reduce the associated mortality rate.

## Introduction

Acute mesenteric ischemia (AMI) is a life-threatening disease that varies in acuity, etiology, presentation, and severity. It requires prompt diagnosis and is characterized by high mortality in an emergency setting [1]. Among the different etiologies of AMI that have been identified (e.g., arterial embolism, arterial thrombosis, venous thrombosis, and nonocclusive mesenteric ischemia), arterial emboli are the most frequent and are responsible for 40% to 50% of cases [2,3]. Infective endocarditis (IE) remains a lethal disease despite advances in medical and surgical care. Systemic embolization, which is a life-threatening complication occurs in 20-50% of patients. The most common sites are the brain and spleen in left-sided IE [4]. Superior mesenteric artery (SMA) septic embolization is an extremely rare and potentially fatal complication of IE. Here, we report a unique case of mitral valve endocarditis prior to emergency cardiac surgery that developed AMI by embolism of a vegetation fragment. Because of its rarity, literature on AMI in the IE setting is scarce. Thus, appropriate treatment strategies are needed to improve its outcomes

## Case presentation

On February 15, 2022, a 38-year-old woman without a known history of cardiac disease was admitted to our emergency department because of intermittent fever, malaise, and a regurgitant mitral murmur. In December 2021, she had a normal spontaneous vaginal delivery, after which she developed a fever and received antibiotics. At admission, the clinical examination revealed a heart rate of 109 beats/min, blood pressure 123/71 mmHg, a respiration rate of 19 breaths/min, temperature 36.4 °C, systolic murmur in the mitral area, and Janeway lesions in her fingers and toes. Table 1 outlines laboratory values at the time of admission and during the hospital stay.

**Table 1:** Laboratory data during hospitalization Hs-CRP, High-sensitivity C-reactive protein; NT-proBNP, N-terminal pro-brain natriuretic peptide.

	At the time of admission	Prior to endovascular revascularization	Ten hours after endovascular revascularization	Three days after mitral valve replacement	Prior second emergency exploratory laparotomy
Hemoglobin (g/dL)	10.2	9.7	11.5	10.3	3.7
Leukocytes (10^3^/µL)	9.97	11.05	10.7	14.4	21.56
Creatinine (mg/dL)	0.56	0.38	0.47	0.34	0.68
Blood glucose level (mg/dL)	83.8	104	150	98.3	68
Hs-CRP (mg/L)	229	226	229	175	111
Blood lactate (mmol/L)	0.6	0.6	1.0	0.7	16
NT-pro-BNP (pg/mL)	447	794	705	943	1300

**Figure 1:**
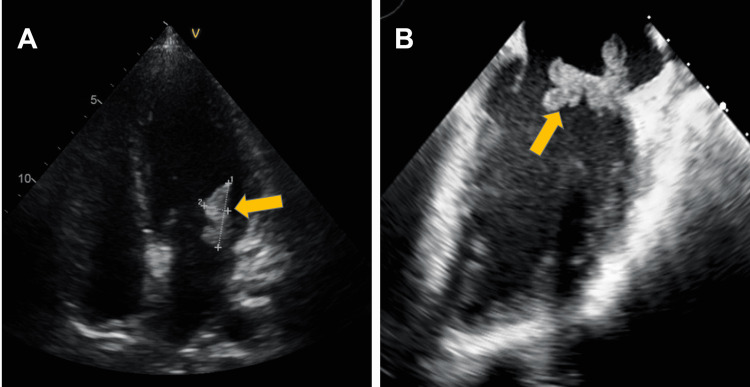
Transthoracic and transesophageal echocardiography (A) Preoperative transthoracic echocardiography demonstrates mobile vegetations on posterior mitral leaflets (arrow). (B) Transesophageal echocardiogram in where a large, mobile vegetation is observed, adhered to the posterior leaflet of the mitral valve.

Baseline laboratory exams were: hemoglobin 10.2 g/dL, leucocytes 9.97x10^3^/mL, neutrophils 8.2x10^3^/mL, C reactive protein 229 mg/L, NT-pro-BNP 447 pg/mL, and blood lactate 0.6 mmol/L. Transthoracic and transesophageal echocardiography revealed multiple mobile 18-24 mm vegetations on both leaflets of the mitral valve with moderate to severe mitral regurgitation (Figure 1).

**Figure 2:**
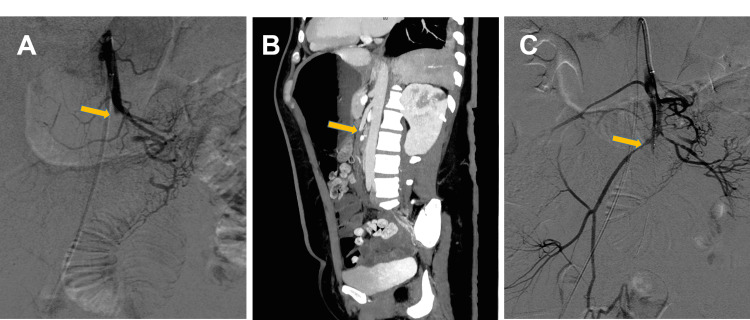
Superior mesenteric arteriography (A) Selective superior mesenteric arteriography shows occlusion proximal to the superior mesenteric artery (SMA). (B) Sagittal view occlusion of SMA. (C) Selective superior mesenteric arteriography after mechanical thrombectomy and thrombolysis demonstrated restored flow in the SMA.

The initial thoraco-abdominopelvic computed tomography (CT) scan and magnetic resonance imaging showed splenic and bilateral renal infarction. The patient was diagnosed with IE of the native mitral valve and severe mitral regurgitation, complicated with renal and splenic embolization. and antibiotic therapy was started (penicillin and amikacin). Due to the evidence of systemic embolisms and the dimensions and mobility of the vegetations, the decision was made to perform emergent mitral valve replacement. Shortly after arrival, the patient developed severe abdominal pain. The patient was immediately examined by a contrast-enhanced CT scan of the abdomen, which revealed contrast filling in the proximal SMA, suggesting thromboembolic occlusion of the SMA. After detecting this complication, anticoagulation and broad-spectrum antibiotics were administrated to reduce the consequences of bacterial translocation (ceftriaxone, amikacin, and metronidazole). At first, the patient was considered for urgent revascularization of the SMA. At the time of angiography, the serum lactate level was 0.6 mmol/L (Table 1). and selective SMA angiography demonstrated occlusion in the proximal region of the SMA (Figures 2A, 2B). Mechanical thrombectomy was performed, a bolus of 10 mg alteplase was administered in situ, and blood flow in the SMA was restored (Figure 2C). Her symptoms improved after the procedure.

Ten hours after the procedure, there was no clinical evidence of intestinal ischemia and the serum lactate level was 1.0 mmol/L (Table 1). However, after discussing the case with the cardiovascular surgeon, the patient underwent mitral valve replacement surgery using a St. Jude Medical Epic bioprosthesis (St Jude Medical Inc., St Paul, MN) without complication.

Three days after mitral valve replacement, the patient complained of abdominal pain. Laboratory analysis revealed leukocytosis of 14,400/L and a lactate level of 0.7 mmol/L (Table 1). CT angiography was performed again, which showed mesenteric thrombosis of the SMA. An emergency exploratory laparotomy was performed, 50-cm resection of the ischemic small intestine combined with an embolectomy of the SMA was performed. However, eight days later, she presented again with signs of an acute abdomen with a lactate level of 16 mmol/L (Table 1), therefore a second emergency exploratory laparotomy was executed. Extensive, deep ischemia of the small and large intestine was found, and a major intestinal resection was performed. After the emergency procedure, her clinical condition worsened, and she developed septic shock and multiple organ failure. Finally, the patient died after 18 days of hospitalization.

## Discussion

We describe an unusual case of a patient who presented with two simultaneous life-threatening pathologies that each required emergent treatment: mitral valve IE requiring emergency surgery and AMI due to embolism in the SMA hours before cardiac surgery. This was a complicated clinical scenario with therapeutic implications.

The American Association for Thoracic Surgery guidelines recommends emergency surgery in patients with left-sided native valve endocarditis because large mobile vegetations on the mitral valve leaflet have been shown to be associated with higher embolic risk [5]. The patient developed an AMI due to embolism prior to surgery, which forced us to suspend cardiac surgery. There is little information on the scenario presented by our patient. Our diagnostic and therapeutic approach through early endovascular treatment allowed the mesenteric ischemic process to be aborted, which then allowed her to successfully undergo mitral valve replacement shortly thereafter. Endovascular treatment including percutaneous aspiration thrombectomy and local fibrinolysis should be considered as soon as possible for acute thrombosis of SMA when the ischemia is potentially reversible, this technique was applied to our patient [6,7]. It is important to emphasize that these interventional procedures can precipitate AMI, favored by extracorporeal circulation, and subsequent in situ thrombotic events. After cardiac surgery, our patient presented again with occlusion of the SMA, for which she required immediate surgical intervention. AMI is a rare complication following open-heart surgery (0.2-0.4%), with mortality rates ranging between 70% and 100% [8].

After the endovascular revascularization on the SMA, at what time should the mitral valve replacement surgery have been performed? Any patient in whom SMA embolization is diagnosed requires early restoration of mesenteric flow to decrease morbidity and mortality. Furthermore, because the most frequent preventable complication is embolism to the brain, the trend tends to be more aggressive in patients with IE and large mobile vegetations requiring early surgery.

## Conclusions

This unusual case highlights the fact that an IE that requires an emergent cardiac surgery, could be complicated by AMI due to embolism prior to surgery, so it becomes a challenge for the cardiovascular surgeon, the anesthesiologist, and the intensive therapy team. Thus, all emphasis must be laid on the prompt patient response, timely and accurate diagnosis, and the design of a better therapeutic strategy to reduce the associated mortality rate.
